# Analysis combining correlated glaucoma traits identifies five new risk loci for open-angle glaucoma

**DOI:** 10.1038/s41598-018-20435-9

**Published:** 2018-02-15

**Authors:** Puya Gharahkhani, Kathryn P. Burdon, Jessica N. Cooke Bailey, Alex W. Hewitt, Matthew H. Law, Louis R. Pasquale, Jae H. Kang, Jonathan L. Haines, Emmanuelle Souzeau, Tiger Zhou, Owen M. Siggs, John Landers, Mona Awadalla, Shiwani Sharma, Richard A. Mills, Bronwyn Ridge, David Lynn, Robert Casson, Stuart L. Graham, Ivan Goldberg, Andrew White, Paul R. Healey, John Grigg, Mitchell Lawlor, Paul Mitchell, Jonathan Ruddle, Michael Coote, Mark Walland, Stephen Best, Andrea Vincent, Jesse Gale, Graham RadfordSmith, David C. Whiteman, Grant W. Montgomery, Nicholas G. Martin, David A Mackey, Janey L. Wiggs, Stuart MacGregor, Jamie E. Craig, The NEIGHBORHOOD consortium, R. Rand Allingham, Murray Brilliant, Donald L. Budenz, John H. Fingert, Douglas Gaasterland, Teresa Gaasterland, Lisa Hark, Michael Hauser, Robert P. Igo, Peter Kraft, Richard K. Lee, Paul R. Lichter, Yutao Liu, Syoko Moroi, Margaret Pericak-Vance, Anthony Realini, Doug Rhee, Julia E. Richards, Robert Ritch, Joel S. Schuman, William K. Scott, Kuldev Singh, Arthur J. Sit, Douglas Vollrath, Gadi Wollstein, Donald J. Zack

**Affiliations:** 1 QIMR Berghofer Medical Research Institute, Brisbane, Queensland Australia; 2 University of Tasmania, Hobart, Tasmania Australia; 3 Population and Quantitative Health Sciences, Institute for Computational Biology, Case Western Reserve University School of Medicine, Cleveland, OH USA; 4 Department of Ophthalmology, Harvard Medical School, Massachusetts Eye and Ear Infirmary, Boston, MA USA; 5 Channing Division of Network Medicine, Brigham and Women’s Hospital, Harvard Medical School, Boston, Massachusetts USA; 6 Department of Ophthalmology, Flinders University, Adelaide, South Australia Australia; 7 South Australian Health & Medical Research Institute, School of Medicine, Flinders University, Adelaide, South Australia Australia; 8 South Australian Institute of Ophthalmology, University of Adelaide, Adelaide, South Australia Australia; 9 Ophthalmology and Vision Science, Macquarie University, Sydney, New South Wales Australia; 10 Department of Ophthalmology, University of Sydney, Sydney, Australia; 11 Centre for Vision Research, The Westmead Institute for Medical Research, University of Sydney, Westmead, NSW Australia; 12 Centre for Eye Research Australia (CERA), University of Melbourne, Royal Victorian Eye and Ear Hospital, Melbourne, Victoria Australia; 13 Department of Ophthalmology, University of Auckland, Auckland, New Zealand; 14 Department of Ophthalmology, University of Otago, Dunedin, Otago New Zealand; 15 School of Medicine, University of Queensland, Herston Campus, Brisbane, QLD Australia; 16 Institute for Molecular Bioscience, The University of Queensland, Brisbane, Queensland Australia; 17 Centre for Ophthalmology and Visual Science, Lions Eye Institute, University of Western Australia, Perth, Australia; 18 Department of Ophthalmology, Duke University Medical Center, Durham, NC USA; 19 Center for Human Genetics, Marshfield Clinic Research Foundation, Marshfield, WI USA; 20 Department of Ophthalmology, University of North Carolina, Chapel Hill, NC USA; 21 Department of Ophthalmology, University of Iowa, College of Medicine, Iowa City, IA USA; 22 Department of Anatomy and Cell Biology, University of Iowa, College of Medicine, Iowa City, IA USA; 23 Eye Doctors of Washington, Chevy Chase, MD USA; 24 Scripps Genome Center, University of California at San Diego, San Diego, CA USA; 25 Wills Eye Hospital, Glaucoma Research Center, Philadelphia, PA USA; 26 Department of Medicine, Duke University Medical Center, Durham, NC USA; 27 Department of Epidemiology and Biostatistics, Institute for Computational Biology, Case Western Reserve University School of Medicine, Cleveland, Ohio, USA; 28 Department of Epidemiology, Harvard School of Public Health, Boston, MA USA; 29 Program in Genetic Epidemiology and Statistical Genetics, Harvard School of Public Health, Boston, MA USA; 30 Bascom Palmer Eye Institute, University of Miami Miller School of Medicine, Miami, FL USA; 31 Department of Ophthalmology and Visual Sciences, University of Michigan, Ann Arbor, MI USA; 32 Department of Cellular Biology and Anatomy, Georgia Regents University, Augusta, GA USA; 33 James & Jean Culver Vision Discovery Institute, Georgia Regents University, Augusta, GA USA; 34 Institute for Human Genomics, University of Miami Miller School of Medicine, Miami, FL USA; 35 Department of Ophthalmology, West Virginia University Eye Institute, Morgantown, WV USA; 36 Department of Ophthalmology and Visual Sciences, UH Cleveland Medical Center, Cleveland, OH USA; 37 Department of Epidemiology, University of Michigan, Ann Arbor, MI USA; 38 Einhorn Clinical Research Center, Department of Ophthalmology, New York Eye and Ear Infirmary of Mt. Sinai, New York, NY USA; 39 Department of Ophthalmology, University of Pittsburgh, Pittsburgh, PA USA; 40 Department of Ophthalmology, Stanford University School of Medicine, Palo Alto, CA USA; 41 Department of Ophthalmology, Mayo Clinic, Rochester, MN USA; 42 Department of Genetics, Stanford University School of Medicine, Palo Alto, CA USA; 43 Wilmer Eye Institute, Johns Hopkins University Hospital, Baltimore, MD USA

## Abstract

Open-angle glaucoma (OAG) is a major cause of blindness worldwide. To identify new risk loci for OAG, we performed a genome-wide association study in 3,071 OAG cases and 6,750 unscreened controls, and meta-analysed the results with GWAS data for intraocular pressure (IOP) and optic disc parameters (the overall meta-analysis sample size varying between 32,000 to 48,000 participants), which are glaucoma-related traits. We identified and independently validated four novel genome-wide significant associations within or near *MYOF* and *CYP26A1*, *LINC02052* and *CRYGS*, *LMX1B*, and *LMO7* using single variant tests, one additional locus (*C9*) using gene-based tests, and two genetic pathways - “response to fluid shear stress” and “abnormal retina morphology” - in pathway-based tests. Interestingly, some of the new risk loci contribute to risk of other genetically-correlated eye diseases including myopia and age-related macular degeneration. To our knowledge, this study is the first integrative study to combine genetic data from OAG and its correlated traits to identify new risk variants and genetic pathways, highlighting the future potential of combining genetic data from genetically-correlated eye traits for the purpose of gene discovery and mapping.

## Introduction

OAG is characterized by optic nerve damage and progressive loss of peripheral vision, with many patients remaining undiagnosed until severe irreversible vision loss has occurred^1,2^. OAG has a significant genetic component with a relative risk of over 9 in first-degree relatives of affected individuals compared to relatives of unaffected people^3^. Our previous genome-wide association studies (GWAS) have reproducibly identified several risk loci for OAG including *TMCO1*, *CDKN2B-AS1, SIX6, CAV1*, *CAV2*, *ABCA1*, *AFAP1*, *GMDS, ARHGEF12*, *TXNRD2*, *ATXN2*, and *FOXC1*^4–9^. However, the majority of the genetic variance contributing to OAG remains unexplained, emphasizing that further studies to identify additional risk loci for OAG are required in order to make genetic risk prediction more clinically useful.

Optic disk parameters including cup area (CA; the central area), disc area (DA; the total area of optic disc including cup area and the surrounding area containing axons of the retinal ganglion cells), and vertical cup-disc ratio (VCDR; the ratio of the vertical diameter of cup area to the vertical diameter of the optic disc) are key measurements used to assess OAG diagnosis and progression^10^. Elevated intraocular pressure (IOP) is the major known risk factor for OAG^2^. We refer to CA, DA, VCDR, and IOP as OAG endophenotypes or quantitative traits. There are high genetic correlations between these quantitative traits and OAG, with several of the already known risk loci for these traits overlapping with each other and with OAG loci, demonstrating their utility as endophenotypes^11–14^. These findings suggest that combining genetic data from OAG and its endophenotypes has the potential to increase the probability of identifying genetic variants that are common between traits, thus enabling the extraction of greater genetic power from valuable disease cohorts.

In this study, we sought to identify additional risk loci contributing to OAG susceptibility by (1) increasing the sample size for OAG, (2) combining GWAS data from OAG and its endophenotypes in order to increase our statistical power to identify new risk loci for OAG, and (3) applying gene and pathway based approaches.

## Results

In total, 3,071 OAG cases from the Australian & New Zealand Registry of Advanced Glaucoma (ANZRAG) obtained in three phases of data collection, and 6,750 unscreened controls of European descent were used as the GWAS discovery dataset in this study (Supplementary Table 1). Five loci were associated with OAG at genome-wide significance level in the meta-analysis of GWAS results between the three phases of ANZRAG data (P < 5 × 10^−8^), including regions near or within *CDKN2B-AS1*, *ABCA1*, *C14orf39* and *SIX6*, *TMCO1*, and *ARHGEF12*, all of which are now well established risk loci for OAG^5–7,9^. Manhattan and Q-Q plots are shown in Supplementary Figure 1. Genomic inflation factor lambda was 1.006 for this analysis.

Next, to increase the power of this study to identify new risk loci for OAG, we performed GWAS meta-analyses of ANZRAG OAG and each of the endophenotypes (CA, DA, VCDR, and IOP) that we obtained from our previous study^11^ (Supplementary Table 1). Before performing the meta-analyses, we confirmed validity of the endophenotypes for OAG by showing that there were significant genetic correlations between OAG and the endophenotypes (ranging between 20% and 47%, Supplementary Table 2; p ≤ 0.018) using the cross-trait bivariate LD score regression approach^15^. By design, the endophenotype studies did not include any of the OAG cases. This was further confirmed in the LD score bivariate analyses where the intercepts were close to zero with 95% confidence intervals (CI) overlapping zero, indicating that there was not significant sample overlap between our OAG and the endophenotypes studies. Moreover, intercepts of the univariate LD score regression analyses^16^ were close to 1 with 95% CIs overlapping 1 (Supplementary Table 3), indicating that there was no model misspecification and other sources of bias such as population stratification and cryptic relatedness in either study^16^.

Four genomic regions that were genome-wide significant in meta-analyses of ANZRAG OAG and one of the endophenotypes (Table 1), and were not previously known risk loci for OAG, and had at least P < 0.05 in the OAG separate analysis, were taken forward for validation. The best SNPs within these regions were rs72815193[G] (risk alleles are indicated within brackets) (P = 6.10 × 10^−10^) on chromosome 10 near *MYOF, XRCC6P1*, and *CYP26A1* for combined OAG and VCDR (in European and Asian ancestries), rs56962872[G] (P = 2.81 × 10^−8^) on chromosome 3 within *LINC02052* and near *CRYGS* for combined OAG and VCDR (in European ancestry), rs6478746[G] (P = 4.54 × 10^−8^) on chromosome 9 near *LOC105376277* and *LMX1B* for combined OAG and CA (in European ancestry), and rs148639588[T] (P = 3.53 × 10^−8^) on chromosome 1 near *COL11A1* for combined OAG and CA (in European ancestry).

**Table 1:** Association results for the best SNPs within the genome-wide significant regions in meta-analyses of ANZRAG OAG and the endophenotypes. Effect sizes of these SNPs on OAG are presented in Table 2.

Chr	SNP	Risk allele	P-value	Analysis	Meta-analysis heterogeneity P	Nearest Genes
10	rs72815193	G	6.10 × 10^−10^	OAG + VCDR	0.31	*MYOF* and *XRCC6P1*
3	rs56962872	G	2.81 × 10^−8^	OAG + VCDR	0.51	*LINC02052* and *CRYGS*
9	rs6478746	G	4.54 × 10^−8^	OAG + CA	0.44	*LOC105376277* and *LMX1B*
1	rs148639588	T	3.53 × 10^−8^	OAG + CA	0.71	*COL11A1*

OAG, open-angle glaucoma; CA, cup area; VCDR, vertical cup to disk ratio.

**Figure 1:**
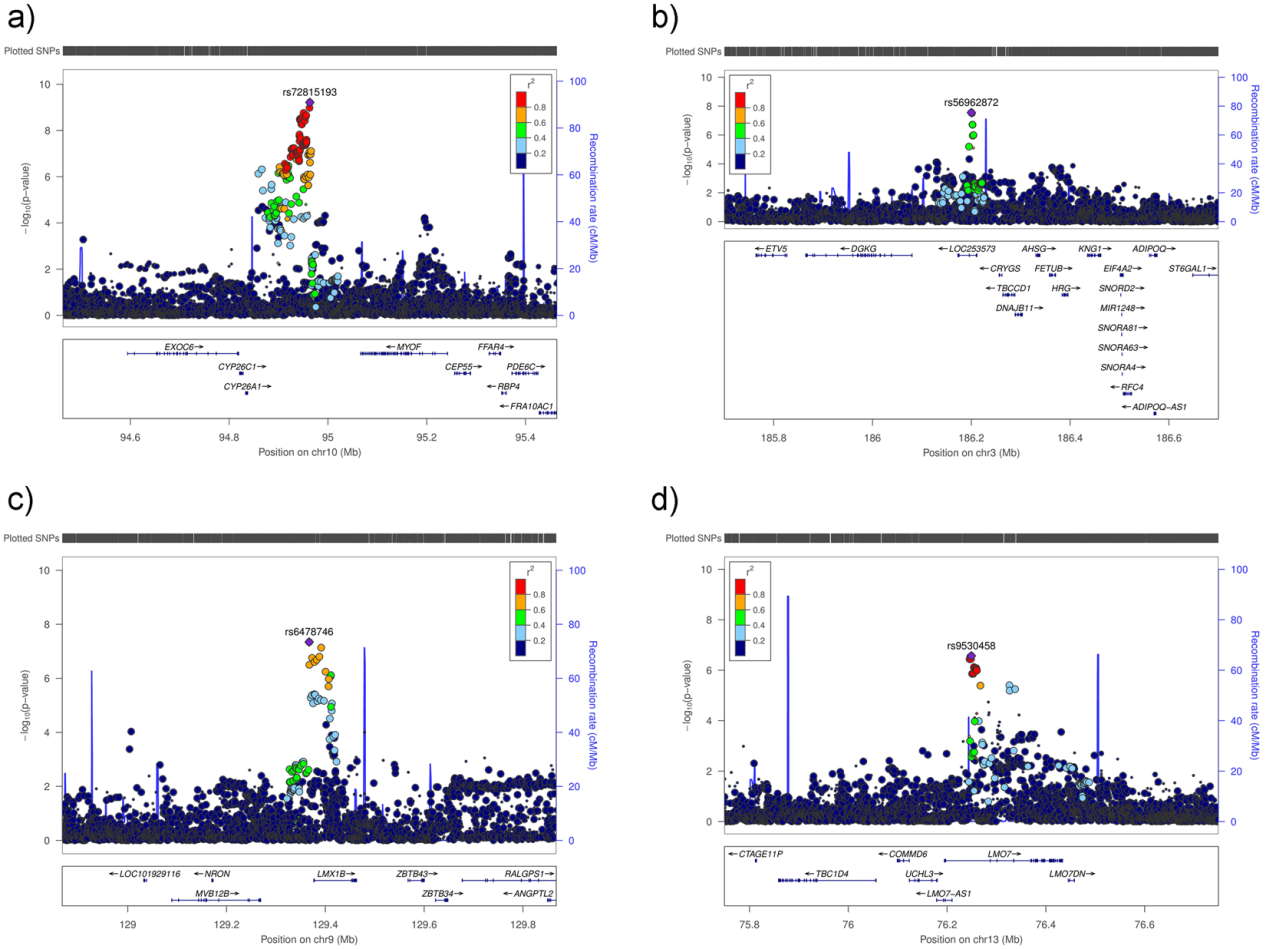
Regional plots for the new risk loci identified in single variant analyses in this study. The most significantly associated SNPs in each region are marked as solid purple diamonds. Pairwise correlations (LD *r*^2^) between the top SNP and the other SNPs in a 400 kb flanking region are illustrated by different colours. Blue spikes show estimated recombination rates. (**a**) rs72815193 on chromosome 10 near *MYOF*, *CYP26A1*, and *CYP26C* (the most significant results were obtained in combined OAG and VCDR analysis conducted in combined Asians and European ancestry). (**b**) rs56962872 on chromosome 3 within *LOC253573* (*LINC02052)*, near *CRYGS* and *TBCCD1* (the most significant results were obtained in combined OAG and VCDR analysis conducted in European ancestry). (**c**) rs6478746 on chromosome nine near *LMX1B* (the most significant results were obtained in combined OAG and CA in European ancestry). (**d**) rs9530458 on chromosome 13 within *LMO7* (combined OAG data from ANZRAG and NEIGHBORHOOD). cM = centimorgan.

Of the above four loci that were genome-wide significant in our discovery meta-analyses, two loci (*LOC105376277*/*LMX1B* and *LINC02052*/*CRYGS)* were replicated (P < 0.0125, the Bonferroni-corrected threshold considering four independent tests) for OAG in an independent replication study, the National Eye Institute Glaucoma Human Genetics Collaboration Heritable Overall Operational Database (NEIGHBORHOOD) (Supplementary Notes), containing 3,853 OAG cases and 33,480 controls^8^ (Table 2). Furthermore, rs72815193 near *MYOF* and *XRCC6P1* had a P = 0.06, and rs4918865 (in high LD with rs72815193; LD r^2^ = 0.93) had a P = 0.02 for OAG in NEIGHBORHOOD. Although the SNPs near *MYOF* and *XRCC6P1* did not pass the Bonferroni-corrected threshold of P < 0.0125, rs4918865 was more strongly associated with the high-tension glaucoma (HTG) subset (P = 0.003 for HTG vs. P = 0.37 for normal-tension glaucoma (NTG)) in NEIGHBORHOOD. The *COL11A1* association was not replicated in NEIGHBORHOOD (P = 0.41 for rs148639588). The statistics including effect sizes of the top SNPs within the three new replicated loci with OAG separately (the meta-analysed OAG data from ANZRAG and NEIGHBORHOOD studies, without including the endophenotype data) are summarized in Table 2. All of the three new replicated loci were associated with CA and VCDR at, at least, nominal significance (P < 0.05), while *LMX1B* was also nominally (P = 0.03) associated with IOP (Table 3). Manhattan and Q-Q plots are shown in Supplementary Figure 1, and regional association plots in Fig. 1. Genomic inflation factor lambda ranged between 1.03 and 1.05 for these analyses.

**Table 2:** GWAS statistics for the new OAG loci in ANZRAG (the discovery OAG set), NEIGHBORHOOD (the replication OAG set), and combined (fixed-effect meta-analysis).

Chr	SNP	Effect allele	Other allele	ANZRAG			NEIGHBORHOOD			combined		
				OR	SE	P-value	OR	SE	P-value	OR	SE	P-value
10	rs4918865^	C	G	1.149	0.03	1.89 × 10^−5^	1.086	0.04	0.01958	1.119	0.02	2.31 × 10^−6^
3	rs56962872	A	G	0.862	0.04	2.91 × 10^−5^	0.892	0.04	0.002324	0.876	0.03	3.03 × 10^−7^
9	rs6478746	A	G	0.853	0.04	1.09 × 10^−5^	0.909	0.04	0.01231	0.879	0.03	9.10 × 10^−7^
13	rs9530458	T	C	1.158	0.03	4.50 × 10^−6^	1.138	0.03	0.00018	1.148	0.02	3.45 × 10^−9^

^rs4918865 in high LD with rs72815193, LD r^2^ = 0.93; OR, odds ratio; SE, standard error of regression coefficent.

**Table 3:** Association of the new loci with OAG and each of the endophenotypes separately.

Chr	SNP	P-value OAG (ANZRAG)	P-value OAG (combined)*	P-value CA	P-value DA	P-value VCDR	P-value IOP	Nearest gene
10	rs4918865^	1.89 × 10^−5^	2.31 × 10^−6^	6.66 × 10^−5^	0.9062	2.10 × 10^−5^	0.3966	*MYOF* and *XRCC6P1*
3	rs56962872	2.91 × 10^−5^	3.03 × 10^−7^	0.000321	0.2832	0.000209	0.3793	*LINC02052* and *CRYGS*
9	rs6478746	1.09 × 10^−5^	9.10 × 10^−7^	0.001659	0.6499	0.003575	0.03146	*LOC105376277* and *LMX1B*
13	rs9530458	4.50 × 10^−6^	3.45 × 10^−9^	0.01305	0.2964	0.01631	0.04911	*LMO7*

^rs4918865 is in high LD with rs72815193, LD r^2^ = 0.93; *Meta-analysis of OAG in ANZRAG and NEIGHBORHOOD data; OAG, open-angle glaucoma; CA, cup area; DA, disk area; VCDR, vertical cup to disk ratio; IOP, intraocular pressure.

Next, results for the SNPs that were not genome-wide significant, but approached this threshold (SNPs with P < 1 × 10^−7^) in the ANZRAG OAG meta-analysis or the meta-analysis of OAG and its endophenotypes, were combined with those from the NEIGHBORHOOD replication data, using a fixed-effects meta-analysis. A fourth locus on chromosome 13 within *LMO7* that was nearly genome-wide significant in the OAG and CA (European ancestry) meta-analysis (rs9530458 [T], P = 2.71 × 10^−7^) became genome-wide significant (rs9530458 [T], OR = 1.148, P = 3.45 × 10^−9^) in the meta-analysis of the OAG data (ANZRAG discovery and NEIGHBORHOOD replication studies), without including the endophenotypes. This SNP was nominally associated with CA, VCDR and IOP (Table 3). Regional association for this locus is plotted in Fig. 1.

We also investigated association of the new loci with NTG and HTG subsets within the ANZRAG and NEIGHBORHOOD data (overall 1,546 NTG cases, 3,412 HTG cases, and 40,230 controls). The results summarized in Supplementary Table 4 show that the 95% CIs overlap between the NTG and HTG analyses, suggesting that these loci may affect both NTG and HTG. However, larger sample sizes are required to further investigate this, as especially for NTG, the 95% CIs are quite wide, and for the *LMO7* SNP (rs9530458) overlaps 1.

We performed a series of sensitivity analyses by excluding the ANZRAG cases in which visual field data was unavailable (585 people) as well as people with mixed-mechanism glaucoma (277 people with OAG as well as a secondary glaucoma) to ensure that the results were not driven by uncertainty in phenotype assignment. The results from the sensitivity analyses in ANZRAG were meta-analysed with the endophenotype or NEIGHBORHOOD results as for the main analysis (Supplementary Tables 5 and 6). Overall, effect sizes obtained from the original and sensitivity analyses were similar, suggesting that our results were not biased by presence of any phenotype uncertainties.

Interestingly, rs72815193 and rs4918865 within the *MYOF* and *XRCC6P1* locus are in high LD (r^2^ = 0.840 and r^2^ = 0.9, respectively) with rs10882165, a SNP that has been shown to be associated with refractive error (P = 1 × 10^−11^)^17^, indicating that this locus may affect glaucoma and its endophenotypes as well as myopia. In addition, SNPs within *LMO7* have been suggestively associated with corneal astigmatism (P = 4 × 10^−6^ for rs11841001)^18^. However, rs9530458 is in low LD (r^2^ = 0.14) with rs11841001 (P = 0.06 in the OAG and CA analysis in Europeans), suggesting that even if the *LMO7* gene affects glaucoma as well as corneal astigmatism, this effect may come from independent risk variants within *LMO7*. On the other hand, the *MYOF* and *XRCC6P1* locus is ~1 Mb away from *PLCE1*, a known risk locus for VCDR. However, rs72815193 (*MYOF*) is not in LD with rs7072574 (*PLCE1*) (LD r^2^ = 0.001) (P = 3.86 × 10^−6^ in the OAG and VCDR analysis in Europeans), suggesting that these are independent loci.

### Gene-based results

We used the approaches implemented in MetaXcan^19^, fastBAT^20^, and EUGENE^21^, to identify genes whose genetic variants or expression levels were significantly associated with development of OAG and its endophenotypes. These gene-based tests are complementary since they make different assumptions and use different approaches and input data to identify associated genes. After Bonferroni correction for multiple testing (refer to the Methods section), nine genes (one from fastBAT, five from MetaXcan, and three from Eugene approaches, Table 4) that were genome-wide significant in the gene-based methods, and were not overlapping with the known risk loci for OAG and its endophenotypes, were taken forward for validation in NEIGHBORHOOD. For MetaXcan and EUGENE approaches, we investigated replication of the significant genes in the same tissues that showed significance in the discovery set since the results from these approaches are tissue-specific.

**Table 4:** Previously unreported genes that were genome-wide significant in gene-based approaches in the discovery datasets, with their corresponding results in the replication dataset (NEIGHBORHOOD).

Genes	P-value	Tissue	Analysis	N_discovery_	Approach	NEIGHBORHOOD replication P-value	N_replication_
*C9*	4.93 × 10^−7^	NA	OAG and IOP: European ancestry	187	fastBAT	0.04	87
*FAM203A*	2.70 × 10^−8^	Nerve_Tibial	OAG and CA: European ancestry	5	MetaXcan	0.14	2
*HERC4*	1.06 × 10^−9^	Cells_Transformed_fibroblasts	OAG and DA: European ancestry and Asians	1	MetaXcan	0.20	1
*RNF26*	2.6 × 106^−9^	Brain_Cerebellum	OAG and IOP: European ancestry and Asians	5	MetaXcan	0.0004	5
*NPAS4*	3.97 × 10^−9^	Uterus	OAG and VCDR: European ancestry and Asians	2	MetaXcan	0.05	2
*CAPN1*	1.02 × 10^−8^	Esophagus_Gastroesophageal_Junction	OAG and VCDR: European ancestry	2	MetaXcan	0.12	2
*DHRS7*	1.00 × 10^−6^	brain	OAG and CA: European ancestry and Asians	1	EUGENE	0.07	1
*HLA-DQA2*	1.00 × 10^−6^	brain	OAG and DA: European ancestry	34	EUGENE	0.70	30
*HLA-DQB1*	1.00 × 10^−6^	brain	OAG and DA: European ancestry	35	EUGENE	0.86	31

NA, Not Applicable (fastBat does use a tissue-specific approach); OAG, open-angle glaucoma; CA, Cup Area; DA, Disc Area; VCDR, Vertical Cup to Disc Ratio; IOP, Intraocular Pressure; N_discovery_, Number of SNP(s) used in the discovery set; N_replication_, Number of SNP(s) used in the replication set.

One previously unreported gene became gene-wide significant (P < 7 × 10^−7^; see the Methods section) in the fastBAT approach (Table 4). This gene, complement factor 9 (*C9*) (P = 4.93 × 10^−7^ in the combined OAG and IOP analysis in European ancestry) was replicated in the NEIGHBORHOOD data (P = 0.04). The best result in the single variant tests for rs56345442, the top SNP within *C9*, was observed in the combined OAG and IOP analysis (P = 4.43 × 10^−6^ for combined European ancestry and Asians, and P = 9.998 × 10^−6^ in European ancestry), suggesting that a larger sample size would be required to detect this association at genome-wide significance threshold in the single variant analysis.

Five previously unreported genes were gene-wide significant (P < 5 × 10^−8^; see the Methods section) in the MetaXcan approach (Table 4), all of which were located within 1 Mb of a previously known locus. Of those genes, association of two genes, *RNF26* (P = 2.66 × 10^−9^ in the discovery set) and *NPAS4 (*P = 3.97 × 10^−9^ in the discovery set), were replicated in the NEIGHBORHOOD data (P = 0.0004 and P = 0.05 for *RNF26 and NPAS4*, respectively). However, four of the eQTL SNPs (rs1893261, rs11823300, rs61898351, and rs11217821) used by MetaXcan to impute the gene expression levels for *RNF26* are in LD r^2^ = 0.3 with rs11827818, located within a previously known locus (near *ARHGEF12*) for IOP and OAG. Repeating the analysis without SNPs in LD r^2^ > 0.2 with rs11827818 (56 SNPs remained out of the original 60 SNPs) led to a non-significant result for this gene (P = 0.85). Similarly, excluding the eQTL SNPs in LD r^2^ > 0.2 with rs7931311, an already known locus near *SCYL1* showed a non-significant association for *NPAS4* (P = 0.82; twenty SNPs remained out of the original 25 SNPs for this analysis). Although these results are valuable for the purpose of fine-mapping of the previously known associations, they suggest that *RNF26* and *NPAS4* are not new risk loci for OAG, but are driven by the eQTL SNPs within the previously known loci.

Three previously unreported genes were gene-wide significant (P < 9 × 10^−6^; see the Methods section) in brain using the EUGENE approach. However, none were replicated in brain using the EUGENE approach applied to the NEIGHBORHOOD data. Despite this, while *DHRS7* was associated at P = 0.07 in brain in NEIGHBORHOOD, there was a stronger association at P = 0.0006 in blood. These data suggestively support that *DHRS7* may also be a risk locus for OAG.

Accordingly, in addition to the risk loci identified in the single variant analyses, our gene-based approaches identified and validated *C9* as an additional new risk locus for OAG. The previously reported OAG loci that also passed the gene-wide significance threshold in the gene-based tests included *TMEM136* in the MetaXcan approach, *AFAP1*, *AFAP1-AS*, *ARHGEF12*, and *TXNRD2* in the EUGENE approach, and *TMCO1*, *ABCA1*, *C9orf53*, *CDKN2A*, *CDKN2B*, *CDKN2B-AS1*, *ARHGEF12*, *TMEM136*, *SIX1*, *SIX4*, *SIX6*, *AFAP1*, *GMDS*, *CAV1*, and *CAV2* in the fastBAT approach (Supplementary Table 7). These data provide further support for these genes being the target genes within the previously reported risk loci for OAG.

### Pathway-based results

Two genetic pathways survived the significance threshold of P < 1 × 10^−6^ and false discovery rate < 0.05 in the pathway-based analysis in DEPICT^22^. One pathway was the “response to fluid shear stress” (GO: 0034405, P = 2.09 × 10^−7^, FDR < 0.01) in the combined OAG and CA analysis, and the other was “abnormal retina morphology” (MP: 0001325, P = 2.50 × 10^−7^, FDR < 0.01) in the combined OAG and VCDR analysis. The “abnormal retina morphology” pathway is interesting because it emphasizes that common risk loci between OAG and VCDR could be functioning through mechanisms related to retinal formation.

### Gene expression

We also investigated the expression of the nearest genes to the best associated SNPs within the new OAG risk loci using RNA sequencing data from relevant human tissues including optic nerve, optic nerve head, retina, ciliary body pars plicata, trabecular meshwork, corneal endothelium, corneal stroma, and corneal epithelium (see Methods section). We observed a higher expression of *LMX1B* in trabecular meshwork, corneal endothelium, and corneal stroma, *MYOF* in trabecular meshwork and corneal epithelium, and *LMO7* in corneal epithelium (Supplementary Figure 2). A relatively higher expression of *LMX1B* and *MYOF* in the trabecular meshwork is interesting because it is consistent with the previous observations for other known OAG genes such as *MYOC* showing high expression profile in the trabecular meshwork^23^. In addition, the *LMX1B* results are also consistent with the results from the GTEx eQTL studies where rs4837100, the SNP in high LD with the top variant in the *LMX1B* locus is an eQTL (P = 7 × 10^−5^) for *LMX1B* in sub-cutaneous adipose tissues^24^, suggesting that risk variants within this locus may alter expression levels of *LMX1B*.

## Discussion

Our study identified four new OAG risk loci in single variant analyses as well as an additional locus using a gene-based approach. Interestingly, some of these new risk loci contribute to risk of other partially correlated eye diseases including age-related macular degeneration (AMD) and myopia (more details below). We also highlighted two genetic pathways associated with the development of OAG, one of which is gene-sets contributing to morphology of retina.

This study highlights the potential of combining genetic data from correlated eye traits for the purpose of gene discovery and mapping. We showed that meta-analysis of GWAS summary statistics from OAG and its correlated traits (VCDR, CA, DA, and IOP) is capable of identifying new risk loci by increasing statistical power. To our knowledge, this is the first study to use an integrative approach for OAG and its endophenotypes to identify new risk loci for OAG. This approach identifies risk variants common between OAG and its correlated traits, while increasing statistical power to detect variants with small effect sizes at the genome-wide significance threshold, which otherwise requires a much larger OAG sample for successful detection.

While all the new risk loci were at least nominally associated with CA and VCDR, none were associated with DA. This suggests that the new loci identified in this study are more likely to influence the size of the central area of the optic disk, rather than the total disc size. There have been some debates on whether the total size of the optic disc is a suitable trait to predict OAG risk and progression^25^. In this study we estimated a much smaller genetic correlation between DA and OAG as compared with genetic correlation between OAG and CA, VCDR, and IOP. In addition, the majority of the genome-wide significant loci in our meta-analyses of OAG and DA showed significant heterogeneity (P < 0.05) between the GWAS results from OAG and DA (data not shown), further suggesting that DA may not be as suitable as CA and VCDR to be used as an endophenotype for POAG.

Bioinformatics functional features of the newly identified risk loci or variants in high LD (r2 > 0.8) with them are discussed in Supplementary Materials. These loci are either quantitative trait loci that regulate the expression of genes within the regions, change sequence motifs for protein binding sites, or are located within DNAase hypersensitivity regions and within regions with enhancer or promoter motifs (Supplementary discussion).

rs72815193 is an intergenic SNP on chromosome 10 and is located near several genes including *XRCC6P1*, *MYOF*, *CYP26A1*, *CYP26C1*, and *EXOC6*. *MYOF* encodes a calcium/phospholipid-binding protein that plays a role in membrane repair of endothelial cells damaged by mechanical stress (http://www.genecards.org/cgi-bin/carddisp.pl?gene=MYOF). *CYP26A1* and *CYP26C1* are involved in regulation of cellular retinoic acid metabolism, eye development, and maturation of vision function by their effect on retina and retinal ganglion cells during the later stages of eye development^26–28^. Interestingly, microdeletion of approximately 363 kb within this region of chromosome 10, which included *CYP26A1*, *CYP26C1*, and *EXOC6*, was reported in three patients affected by non-syndromic bilateral and unilateral optic nerve aplasia in a Belgian pedigree^29^. Moreover, this locus is also a risk locus for refractive error, where rs10882165 (P = 1 × 10^−11^ for refractive error) is in high LD with rs72815193 (r^2^ = 0.84), the top risk SNP within this locus^17^. *XRCC6P1* is a pseudogene with limited data available on function of this gene or its relevance to diseases.

rs56962872 on chromosome 3 is an intronic variant within the *LOC253573* (*LINC02052)* gene, near *CRYGS* and *TBCCD1*. *LINC02052* is highly expressed in retina and vitreous humor *CRYGS* is a member of the crystallin gene families, which are expressed in human lens, retina, and cornea^30^. Mutations in *CRYGS* are associated with autosomal dominant paediatric cortical cataract in humans^31^. *TBCCD1* is a centrosomal protein that plays a role in the regulation of centrosome and Golgi apparatus positioning, with consequences on cell shape and cell migration^32^. Interestingly, human XRP2 is a TBCC-domain containing protein mutated in certain forms of retinitis pigmentosa, a retinal degenerative disease^33,34^. Thus, TBCC-domain containing proteins including TBCCD1 may play a role in OAG through their effect on retinal formation or mechanisms such as cell shape and function.

rs6478746 on chromosome 9 is located near *LMX1B* and *LOC105376277*. *LMX1B* is mutated in Nail-Patella Syndrome, characterized by nail, patella and elbow dysplasia, in which some patients develop OAG^35^. In support of this, a mouse model study showed that a dominant-negative mutation of *Lmx1b* causes glaucoma^36^. This gene is required for murine trabecular meshwork formation and thus has an important role in controlling IOP^37^, suggesting that this gene may influence OAG through the mechanisms related to increased eye pressure. In support of this, rs6478746 was nominally associated with IOP (P = 0.03), and associated at P = 6.38 × 10^−7^ in the combined OAG and IOP analysis in European ancestry.

rs9530458 is an intronic variant within *LMO7*, a protein-coding gene that may be involved in protein-protein interaction (http://www.genecards.org/cgi-bin/carddisp.pl?gene=LMO7&keywords=LMO7). An engineered 800 kilobase deletion of Uchl3 and Lmo7 caused defects in viability, postnatal growth and degeneration of muscle and retina in mice^38^. In addition, *LMO7* has been suggestively (P = 4 × 10^−6^ for rs11841001) associated with corneal astigmatism. However, rs9530458 is not in high LD with the top corneal astigmatism SNP (rs11841001, LD r^2^ = 0.14, P = 0.06 in the OAG and CA analysis in Europeans), suggesting that independent variants within this gene may be involved in the development of OAG and corneal astigmatism.

*C9*, the gene identified in the gene-based approaches in this study also has interesting implications for OAG. This gene is one component of the complement system, a part of the innate immune response whose deregulation is considered to have a major role in pathogenesis of AMD^39^. Common and rare variants in multiple complement genes including C9 have been associated with AMD^40–42^, consistent with studies showing significant genetic correlation between AMD and glaucoma^43^. Moreover, there is some evidence that the complement system including C9 is activated in glaucomatous optic nerve head astrocytes^44^, suggesting a possible role of *C9* in the development of OAG.

This study has several limitations. First, we did not use the recently proposed approaches for meta-analysis of correlated traits using GWAS summary statistics^45^ which adjust for overlapping or related subjects, population stratification, and heterogeneity of effect between studies. In our study, as confirmed with the LD score regression analyses, we did not have biases such as population stratification and sample overlap between the OAG and endophenotype studies. Thus, we did not use the proposed approaches that adjust for such biases in this study. In addition, approaches such as that proposed by Zhu and colleagues are susceptible to detecting association for a trait that is mainly contributed to via only a subset of the traits. Although our approach has a similar limitation, we investigated the heterogeneity of association between studies to ensure that the results were not biased towards one study. Another limitation of this study is that we performed the combined analysis of OAG and each endophenotype separately, rather than including all the endophenotypes in the same analysis. This was justified due to two reasons: (1) IOP and VCDR loci act through two distinct pathways (intraocular pressure vs optic disc morphology), and (2) the GWAS results for the endophenotypes included in this study were obtained from the same consortia study^11^, and thus the subjects overlap substantially between these phenotypes.

In conclusion, this study highlighted several novel genes and cellular pathways likely to be involved in the development of OAG. Fine-mapping and functional validation of the new risk loci will help to better understand disease pathophysiology. Identification of additional risk loci using larger sample sizes in the future may lead to more accurate genetic risk prediction algorithms for OAG as well as identification of new molecular targets for prevention and intervention strategies.

## Methods

### Study design and participants

In total 3,071 OAG cases from the Australian & New Zealand Registry of Advanced Glaucoma (ANZRAG)^46^, and 6,750 unscreened controls of European descent were included in this study. This dataset involves three phases of OAG data collection, and hence, quality control (QC), imputation, and association analysis were conducted separately for each phase before combining the results in a meta-analysis. The first phase was previously published and comprises 1,155 advanced OAG cases and 1,992 controls genotyped on Illumina Omni1M or OmniExpress arrays (Illumina, San Diego, California, USA)^7^. The second phase includes a further 579 advanced OAG cases genotyped on Illumina HumanCoreExome array and 946 controls selected from parents of twins previously genotyped on the same array. The third phase comprises 1,337 OAG cases (11 advanced, 741 non-advanced, and 585 cases with visual field data unavailable) genotyped on Illumina HumanCoreExome array and 3,812 controls selected from a study of endometriosis previously genotyped on the same array. The diagnostic criteria have been described previously^7^. The Approval was obtained from the Human Research Ethics Committees of Southern Adelaide Health Service/Flinders University, University of Tasmania, QIMR Berghofer Medical Research Institute and the Royal Victorian Eye and Ear Hospital. Written informed consent was obtained from all participants. All the methods were carried out in accordance with relevant guidelines and regulations for human subject research, in accordance with the Declaration of Helsinki.

For the endophenotypes we used GWAS results from our previously published data, which includes varying numbers of participants for each trait; between 22,000 and 24,000 Europeans, and between 7,000 and 9,000 Asians^11^ (Supplementary Table 1). By design, the endophenotype studies did not include any of the OAG cases. We combined the OAG GWAS results with the results obtained from the endophenotype GWASs in Europeans in the primary analysis as well as those obtained from combined European and Asian endophenotype GWASs in a secondary analysis. The QC, imputation, and association testing has been previously described for these studies^11^ as well as for the first phase of the ANZRAG OAG study^7^– specifically imputation was done using the 1000 Genomes Phase 1 Europeans reference panel. The following paragraphs provide this information for the second and third phases of the ANZRAG OAG dataset.

### Quality Control (QC)

We used the same QC protocol as was used for the first phase of the ANZRAG OAG GWAS. Briefly, we performed QC using PLINK 1.9^47,48^ by removing individuals with more than 3% missing genotypes, and SNPs with call rate less than 97%, minor allele frequency (MAF) < 0.01, and Hardy-Weinberg equilibrium P < 0.0001 in controls and P < 5 × 10^−10^ in cases. The same QC protocol was used for case and control datasets before merging to avoid mismatches between the merged datasets. We used PLINK1.9 to compute identity by descent based on autosomal markers, with one of each pair of individuals with relatedness of greater than 0.2 removed within each phase of the ANZRAG data as well as between the three phases. PLINK 1.9 was used to compute principal components for all participants and reference samples of known northern European ancestry (1000 Genomes British, CEU, Finland participants). Participants with PC1 or PC2 values > 6 standard deviations from the mean of known northern European ancestry group were excluded.

### Imputation

Phasing of the genotyped SNPs was conducted using ShapeIT^49^ and imputation was performed using Minimac3 through the Michigan Imputation Server^50^, with the Haplotype Reference Consortium (HRC)^51^ r1.1 as the reference panel. SNPs with imputation quality (r^2^) > 0.3 and MAF > 0.01 were carried forward for analysis.

### Association testing

We assessed associations between SNPs and OAG status adjusted for sex and the first six principal components under an additive genetic model using the dosage scores obtained from imputation. Association analysis was performed either using SNPTEST v2.5^52,53^ or PLINK 1.9. Genomic inflation factor lambda was calculated to investigate the presence of inflation due to model miss-specification or population stratification. We also performed a sensitivity analysis by excluding the OAG cases in which visual field data was unavailable to ensure that the association results were not driven by including those people in the analysis. Similarly, people with mixed-mechanism glaucoma (277 people with OAG as well as a secondary glaucoma) were excluded in a sensitivity analysis as a further robustness check. Association of the top loci were also investigated in NTG and HTG subsets within the ANZRAG dataset (821 NTG cases, 1,544 HTG cases, and 6,750 controls).

To increase the power of this study to identify new risk loci for OAG, we meta-analysed the OAG GWAS results with those obtained from the endophenotype GWASs. To confirm the validity of the endophenotypes for OAG, we estimated genetic correlation between OAG and the endophenotypes using the cross-trait bivariate LD score regression approach^15^. This approach estimates genetic correlation between traits from regression of the combined Z scores of each SNP for two traits obtained from GWAS summary statistics on LD scores calculated from a reference panel. LD scores are incorporated in estimation of genetic correlation based on the fact that SNPs with high LD have, on average, higher chi-square statistics for association with a trait as compared with SNPs with low LD. In addition, an intercept close to zero in these analyses indicates that there is not a significant sample overlap between studies. Moreover, we used the univariate LD score regression approach^16^ to investigate presence of model or structural bias in the OAG and endophenotype GWAS data. An intercept close to 1 in a univariate analysis indicates that there is no model misspecification and other sources of bias such as population stratification and cryptic relatedness^16^.

Meta-analysis of the ANZRAG OAG results between the three phases was performed in METAL^54^ using the fixed-effects inverse-variance weighting approach using SNP effect sizes and their standard errors. In addition, the quantitative trait GWAS results were meta-analysed with the ANZRAG OAG GWAS using the P-value approach in METAL. In this approach, Z scores are created for each SNP from P-values and direction of effect for tested alleles, and combined as weighted sum of the individual statistics where the weights are proportional to the square root of the number of individuals examined in each study. Genomic control correction was applied to each GWAS dataset prior to the meta-analysis to ensure that inflation was not driving our results. We also investigated the heterogeneity of Z scores between studies using the approach implemented in METAL. Q-Q and Manhattan plots were created in R. For the purpose of creating these plots, we excluded genome-wide significant SNPs that showed heterogeneity of effect (Cochran’s Q Test P < 0.05) between OAG and the quantitative traits that included Asians. Regional association plots were created using LocusZoom^55^.

SNPs with P < 1 × 10^−7^ from the overall meta-analysed results that were previously unreported for OAG, and were at least nominally associated (P < 0.05) with OAG in the combined OAG and the quantitative trait analyses, were taken forward for validation in an independent US dataset, the National Eye Institute Glaucoma Human Genetics Collaboration Heritable Overall Operational Database (NEIGHBORHOOD), containing 3,853 OAG cases and 33,480 controls^8^. More details on the NEIGHBORHOOD study has been provided in the Supplementary Notes.

### Gene-based tests

Gene-based tests were conducted using the approaches implemented in MetaXcan^19^ fastBAT^20^, and EUGENE^21^. We used the GWAS results from OAG as well as combined OAG and its endophenotypes for the gene-based tests. MetaXcan is an extension of PredixCan^56^, a gene-based approach that uses GWAS summary results to impute the genetic component of gene expression in different tissues (thus eliminating the need to directly measure gene expression levels), and correlates the imputed gene expressions with phenotypes of interest. The Bonferroni-corrected threshold for multiple testing was set to 5 × 10^−8^, considering the maximum number of 7,230 genes tested in 44 tissues for three traits, OAG, IOP, and VCDR (note that VCDR is the ratio of CA to DA, so highly correlated with these traits). The MetaXcan method is developed based on the publically available European reference data; however, this method is quite robust to ethnicity differences^19^. Thus, we ran the MetaXcan analyses using European ancestry as well as combined Asians and European ancestry data. The combined ethnicity dataset was >80% European.

fastBAT (fast and flexible set-Based Association Test) is a gene-based approach that calculates the association p-values for a set of SNPs (within ±50 Kb of a gene for this study) using GWAS summary data while accounting for LD between SNPs. The Bonferroni-corrected significance threshold was set to 7 × 10^−7^, considering the maximum number of 24,654 genes tested for three traits. We ran the fastBAT analyses using European ancestry and Asians data separately, and combined P-values using the sum of Z scores method. In addition, we also used the combined Asians and European ancestry meta-analysis results as input for this analysis.

EUGENE is a gene-based approach that captures the aggregate effects of independent eQTL SNPs (both *cis*-acting and *trans*-acting) for each gene using GWAS summary statistics. The most suitable tissue for OAG that is available to use with the EUGENE approach is the brain tissue. Considering the maximum number of 5,487 genes tested in brain for three traits, the Bonferroni-corrected threshold was set to 9 × 10^−6^. Since the current version of EUGENE is developed based on publically available European reference data, we ran the EUGENE analyses using the meta-analysis results from subjects with European ancestry only. However, since the combined ethnicity analyses comprised mainly (at least 80%) Europeans, we also ran these analyses using combined Asian and European ancestry data.

### Pathway-based tests

We used the results from the ANZRAG OAG meta-analysis as well as the meta-analysis of ANZRAG OAG and its endophenotypes to do a pathway analysis using the approach implemented in DEPICT^22^. Although it is preferable to use genome-wide significant loci for DEPICT provided there are at least 10 independent risk loci available for a trait, because we did not have this many independent genome-wide significant loci for each of the meta-analyses we used SNPs with P < 1 × 10^−7^ for the pathway analyses. Due to the polygenic nature of the studied traits, as well as our relatively low statistical power to detect SNPs with small effect sizes, including more associated SNPs in the analysis may result in improved power to detect associated pathways. Assuming that all the 14,463 pathways used by DEPICT are independent, and considering testing those pathways for three traits, we set the Bonferroni-corrected significance threshold to P < 1 × 10^−6^ and false discovery rate <0.05.

### Gene expression

Ocular tissues of interest (corneal epithelium, corneal stroma, corneal endothelium, trabecular meshwork, pars plicata of the ciliary body, retina, optic nerve head and optic nerve) were collected from donor human eyes within 24 hours post-mortem (mean = 9.7 ± 5.3 hours) and fixed in RNAlater. RNA quality was assessed using Agilent Bioanalyzer 2100 RNA 6000 Nano Assay (Catalog #G2938C, Santa Clara, USA) (mean RNA integrity number = 6.5 ± 1.8) and concentrations were quantified on the Qubit® 2.0 Fluorometer (Catalog #Q32866, Carlsbad, USA) using Qubit™ RNA Assay Kits (Catalog #Q32852, Carlsbad, USA). 250 nanograms of total RNA from each tissue sample was indexed using Bioo Scientific® NEXTflex™ Rapid Directional mRNA-Seq Kit Bundle with RNA-Seq Barcodes and poly(A) beads (Catalog #5138-10, Austin, Texas) and sequenced on the Illumina NextSeq® 500 using High Output v2 Kit (75 cycles) (Catalog #FC-404-2005, San Diego, USA). All raw sequences were quality-control filtered and trimmed with Trimgalore v0.4.0 (http://www.bioinformatics.babraham.ac.uk/projects/trim_galore/), aligned to the human genome (GRCh38 assembly) using TopHat v2.1.1^57,58^ and normalized using the trimmed mean of M-values (TMM) normalisation method^59^ in Bioconductor R package EdgeR v3.10.2^60^. Gene differential expression was analysed using EdgeR software with Benjamini Hochberg false-positive adjustment^61^.

### *In silico* functional analyses

Bioinformatics functional analyses were performed for the novel genome-wide significant loci using HaploReg^62^, RegulomeDB^63^, ENCODE Project Consortium^64^, and eQTL-browsers including Blood eQTL-Browser^65^ and GTEx-Browser^24^. The top SNP in each locus as well as those with LD r^2^ > 0.8 with the top SNPs were used for these analyses.

### Data availability

The datasets generated during and/or analysed during the current study are not publicly available due to ethical issues.

## Electronic supplementary material

Supplementary Materials
