# Supplementary material for: Analysis combining correlated glaucoma traits identifies five new risk loci for open-angle glaucoma
Source: Sci Rep. 2018 Feb 15;8:3124. doi: 10.1038/s41598-018-20435-9 (PMC5814451; doi:10.1038/s41598-018-20435-9)
Supplement: Supplementary file 1 — Supplementary Materials [file 41598_2018_20435_MOESM1_ESM.pdf]

Analysis combining correlated glaucoma traits identifies five new risk loci for open-angle glaucoma

Puya Gharahkhani, Kathryn P. Burdon, Jessica N. Cooke Bailey, Alex W. Hewitt, Matthew H. Law, Louis R. Pasquale, Jae H. Kang, Jonathan L. Haines, Emmanuelle Souzeau, Tiger Zhou, Owen M. Siggs, John Landers, Mona Awadalla, Shiwani Sharma, Richard A Mills, Bronwyn Ridge, David Lynn, NEIGHBORHOOD consortium, Robert Casson, Stuart L Graham, Ivan Goldberg, Andrew White, Paul R. Healey, John Grigg, Mitchell Lawlor, Paul Mitchell, Jonathan Ruddle, Michael Coote, Mark Walland, Stephen Best, Andrea Vincent, Jesse Gale, Graham RadfordSmith, David C. Whiteman, Grant W. Montgomery, Nicholas G. Martin, David A Mackey, Janey L. Wiggs, Stuart MacGregor, Jamie E. Craig

Supplementary Notes:

National Eye Institute Glaucoma Human Genetics Collaboration Heritable Overall Operational Database (NEIGHBORHOOD) study

All cases and controls met the clinical criteria previously described[1-3]. Subjects were enrolled using a protocol approved by the Massachusetts Eye and Ear Infirmary institutional review board and all subjects signed consent forms approved by the local IRB prior to enrolling in the study. Briefly, Primary Open-angle glaucoma (POAG) cases were defined as individuals for whom reliable visual field (VF) tests showed characteristic VF defects consistent with glaucomatous optic neuropathy. Individuals were classified as affected if the VF defects were reproduced on a subsequent test or if a single qualifying VF was accompanied by a cup-disc ratio (CDR) of 0.7 or more in at least one eye. The majority of cases (over 90%) met this definition, including 96% of the NEIGHBOR cases[2]; and all of the Mass Eye and Ear, NHS, HPFS, and WGHS cases. A small percentage (less than 10%) of the NEIGHBOR, Mayo, Marshfield and Iowa cases were defined by cup-to-disc ratio only because visual field data was not available, in some cases because of advanced disease (poor visual acuity) or other medical condition. The CDR definition was > 0.7 in both eyes or CDR asymmetry between the

two eye of 0.2. In the OHTS study an alternative case definition based on progression of optic nerve degeneration was also used[4] (see below). Patients with signs of secondary causes for elevated IOP such as exfoliation syndrome or pigment dispersion syndrome or critically narrow filtration structures were excluded. Elevation of IOP was not a criterion for inclusion of cases or controls; however 1,868 cases did have a history of elevated IOP (≥22 mm Hg) measured in a clinical setting (typically between the hours of 8AM and 5PM) and were classified as high tension glaucoma (HTG), while 725 cases did not have elevated IOP and were classified as normal tension glaucoma (NTG). For 1,260 cases peak IOP data was not available. The controls were selected to be representative of the age range and gender of the cases. While the average age of cases and controls was not statistically different for any dataset included in the NEIGHBORHOOD, some datasets included cases and controls younger than age 55 which could reduce the power of the study. Controls had IOP < 21 mmHg, as measured in a clinical setting, CDR of less than 0.6 and did not have a family history of glaucoma.

Imputed genotypes (1000 Genomes panel, March 2012) for 3,853 cases and 33,480 controls from 8 independent datasets were used as the discovery cohort for the NEIGHBORHOOD genome-wide association study for POAG[3]. Quality-control was performed for each data set as described in Bailey et al., 2016[3]. Overall sample and genotype call rates were ≥ 95% for each site. Samples with Log R ratio (LRR) and B allele frequency (BAF) values suggestive of copy number variants were removed prior to analysis. Principal components (eigenvectors) were computed for all participants using EIGENSTRAT[5]. For each dataset logistic regression was performed in ProbABEL[6] for all analyses (POAG overall, HTG, NTG), controlling for age, sex, and study-specific covariates including study-specific eigenvectors. Each analysis was evaluated separately for overall genomic inflation (implementing the R package GenABEL) (λ-value ≤ 1.05 for each dataset)[3]. Results were meta-analyzed in METAL[7] implementing the inverse variance weighted method and applying genomic control correction.

Supplementary Tables

Supplementary Table 1. Study overview of the ANZRAG OAG and the endophenotype data.

Study	Number of participants	male %	Number of cases	Number of controls	Genotyping Array	Imputation	reference
ANZRAG OAG (Phase 1)	3,147	58%	1,155	1,992	Illumina Omni1M or OmniExpress	1000G phase1	Gharahkhani et al. 2014[8]
ANZRAG OAG (Phase 2)	1,525	44%	579	946	Illumina HumanCoreExome	HRC r1.1	Not published
ANZRAG OAG (Phase 3)	5,149	26%	1,337	3,812	Illumina HumanCoreExome	HRC r1.1	Not published
CA (Europeans)	22,489	41%	NA	NA	Various Illumina and Affymetrix arrays	1000G phase1	Springelkamp et al. 2017[9]
CA (Asians)	7,339	47%	NA	NA	Various Illumina and Affymetrix arrays	1000G phase1	Springelkamp et al. 2017
DA (Europeans)	22,504	42%	NA	NA	Various Illumina and Affymetrix arrays	1000G phase1	Springelkamp et al. 2017
DA (Asians)	7,307	47%	NA	NA	Various Illumina and Affymetrix arrays	1000G phase1	Springelkamp et al. 2017
VCDR (Europeans)	23,899	41%	NA	NA	Various Illumina and Affymetrix arrays	1000G phase1	Springelkamp et al. 2017
VCDR (Asians)	8,373	49%	NA	NA	Various Illumina and Affymetrix arrays	1000G phase1	Springelkamp et al. 2017
IOP (Europeans)	29,578	42%	NA	NA	Various Illumina and Affymetrix arrays	1000G phase1	Springelkamp et al. 2017
IOP (Asians)	8,352	48%	NA	NA	Various Illumina and Affymetrix arrays	1000G phase1	Springelkamp et al. 2017

NA, Not Applicable; OAG, open-angle glaucoma; CA, Cup Area; DA, Disc Area; VCDR, Vertical Cup to Disc Ratio; IOP, Intraocular Pressure.

Supplementary Table 2. Cross-trait bivariate LD score regression between OAG and the endophenotypes used in this study.

Phenotype one	Phenotype two	Genetic correlation (SE)	P-value*	Intercept (SE)^
OAG	CA	0.4701 (0.070)	2.19e-11	-0.0001 (0.0048)
OAG	DA	0.2026 (0.086)	1.80e-02	-0.0046 (0.0051)
OAG	VCDR	0.4247 (0.069)	5.78e-10	0.0032 (0.0049)
OAG	IOP	0.3917 (0.097)	5.77e-05	0.0031 (0.0048)

OAG, open-angle glaucoma; CA, Cup Area; DA, Disc Area, VCDR, Vertical Cup to Disc Ratio; IOP, Intraocular Pressure; SE, Standard Error. ^An intercept close to zero in a bivariate analysis indicates that there is not a significant sample overlap between two studies.

Supplementary Table 3. Univariate LD score regression for OAG and the endophenotypes used in this study.

Phenotype	Heritability	SE of Heritability	Intercept	SE of Intercept
OAG	0.3362*	0.0446	0.9956	0.0065
CA	0.2683	0.0345	1.0044	0.0076
DA	0.2756	0.0483	1.0089	0.0089
VCDR	0.3173	0.0376	1.0128	0.0083
IOP	0.1603	0.0268	1.0148	0.0079

CA, Cup Area; DA, Disc Area, VCDR, Vertical Cup to Disc Ratio; IOP, Intraocular Pressure; SE, Standard Error. *SNP heritability is reported on the liability scale for OAG.

Supplementary Table 4. Associations of the top SNPs within the new OAG risk loci with NTG and HTG separately. This table reports the results for the combined ANZRAG and NEIGHBORHOOD data (meta-analysis) using 1,546 NTG cases, 3,412 HTG cases, and 40,230 controls.

SNP	Effect allele	Other allele	NTG OR (95% confidence interval)	HTG OR (95% confidence interval)
rs72815193	A	G	0.8662 (0.7967-0.9418)	0.872 (0.8197-0.9277)
rs56962872	A	G	0.8345 (0.7616-0.9144)	0.8862 (0.8287-0.9477)
rs6478746	A	G	0.8224 (0.7513-0.9004)	0.8874 (0.8288-0.9500)
rs9530458	T	C	1.0793 (0.9933-1.1727)	1.1845 (1.1141-1.2593)

NTG, Normal tention glaucoma; HTG, high tention glaucoma; OR, odds ratio.

Supplementary Table 5. Sensitivity analysis by removing OAG cases in which visual field data was unavailable. Odds ratios are reported from the case-control OAG analysis in the ANZRAG study, and P values are reported from meta-analysis of the OAG results with its endophenotypes or the NEIGHBORHOOD replication study.

SNP	Effect allele	Nearest genes	Main analysis OR (95% CIs)	Sensitivity analysis OR (95% CIs)	Main meta-analysis P-value	Sensitivity meta-analysis P-value
rs6478746	A	*LMX1B*	0.853 (0.916-0.795)	0.823 (0.889-0.762)	4.54×10^{-8} (OAG + CA)	4.72×10^{-9} (OAG + CA)
rs56962872	A	*LINC02052*	0.862 (0.924-0.804)	0.844 (0.910-0.782)	2.81×10^{-8} (OAG + VCDR)	1.42×10^{-8} (OAG + VCDR)
rs72815193	A	*MYOF/XRCC6P1*	0.870 (0.927-0.816)	0.847 (0.907-0.791)	6.10×10^{-10} (OAG + VCDR)	5.49×10^{-11} (OAG + VCDR)
rs9530458	T	*LMO7*	1.158 (1.088-1.233)	1.144 (1.069-1.224)	3.45×10^{-9} (OAG discovery + replication)	7.02×10^{-8} (OAG discovery + replication)

OAG, open-angle glaucoma; CA, Cup Area; VCDR, Vertical Cup to Disc Ratio; OR, odds ratio; CI, confidence interval.

Supplementary Table 6. Sensitivity analysis by removing cases with secondary glaucoma. This table shows associations of the top SNPs within the new OAG risk loci after removing 227 people with mixed-mechanism glaucoma (i.e. people who have OAG as well as a secondary glaucoma) from the analysis. Odds ratios are reported from the case-control OAG analysis in the ANZRAG study, and P values are reported from meta-analysis of the OAG results with its endophenotypes or the NEIGHBORHOOD replication study.

SNP	Effect Allele	Nearest genes	Main analysis OR (95% CIs)	Sensitivity analysis OR (95% CIs)	Main meta-analysis P-value	Sensitivity meta-analysis P-value
rs6478746	A	*LMX1B*	0.853 (0.916-0.795)	0.854 (0.918-0.795)	4.54×10^{-8} (OAG + CA)	6.23×10^{-8} (OAG + CA)
rs56962872	A	*LINC02052*	0.862 (0.924-0.804)	0.861 (0.924-0.802)	2.81×10^{-8} (OAG + VCDR	1.32×10^{-8} (OAG + VCDR)
rs72815193	A	*MYOF/XRCC6P1*	0.870 (0.927-0.816)	0.873 (0.931-0.818)	6.10×10^{-10} (OAG + VCDR	1.05×10^{-9} (OAG + VCDR)
rs9530458	T	*LMO7*	1.158 (1.088-1.233)	1.160 (1.088-1.236)	3.45×10^{-9} (OAG discovery + replication)	4×10^{-9} (OAG discovery + replication)

OAG, open-angle glaucoma; CA, Cup Area; VCDR, Vertical Cup to Disc Ratio; OR, odds ratio; CI, confidence interval.

Supplementary Table 7. The previously reported genes for OAG that were also genome-wide significant in the gene-based approaches used in this study.

Genes	Best P-value	Tissue	Analysis	Number of SNPs used	Approach
CDKN2B	4.26×10^{-39}	NA	OAG + CA	86	fastBAT
CDKN2B-AS1	3.24×10^{-38}	NA	OAG + VCDR	246	fastBAT
CDKN2A	3.12×10^{-35}	NA	OAG + VCDR	107	fastBAT
C9orf53	1.88×10^{-31}	NA	OAG + VCDR	100	fastBAT
ABCA1	1.25×10^{-13}	NA	OAG + IOP	441	fastBAT
TMCO1	6.85×10^{-13}	NA	OAG + IOP	163	fastBAT
ARHGEF12	1.55×10^{-12}	NA	OAG + IOP	251	fastBAT
SIX6	7.95×10^{-11}	NA	OAG + CA	67	fastBAT
TMEM136	9.83×10^{-10}	NA	OAG + IOP	113	fastBAT
SIX1	2.33×10^{-9}	NA	OAG + CA	90	fastBAT
CAV1	1.84×10^{-8}	NA	OAG + IOP	142	fastBAT
CAV2	2.18×10^{-8}	NA	OAG + IOP	104	fastBAT
AFAP1	3.31×10^{-8}	NA	OAG + IOP	607	fastBAT
SIX4	1.08×10^{-7}	NA	OAG + CA	101	fastBAT
GMDS	2.10×10^{-7}	NA	OAG + IOP	852	fastBAT
TMEM136	4.25×10^{-11}	TW_Skin_Sun_Exposed_Lower_leg	OAG + IOP	1	MetaXcan
AFAP1	1.00×10^{-6}	Brain	OAG + IOP	1	EUGENE
AFAP1-AS	1.00×10^{-6}	Brain	OAG + CA/IOP/VCDR	2	EUGENE
ARHGEF12	1.00×10^{-6}	Brain	OAG + DA/IOP	1	EUGENE
TXNRD2	1.00×10^{-6}	Brain	IOP	2	EUGENE

NA, Not Applicable (fastBat does use a tissue-specific approach); OAG, open-angle glaucoma; CA, Cup Area; DA, Disc Area; VCDR, Vertical Cup to Disc Ratio; IOP, Intraocular Pressure.

Supplementary Figures

Supplementary Figure 1. Manhattan and Q-Q plots. This figure shows the Manhattan and Q-Q plots for the meta-analysis of OAG between the three phases of ANZRAG (please see the main text) as well as combined OAG and the quantitative traits (VCDR, CA, DA, IOP). Manhattan plots on the left: The SNPs have been plotted against their

chromosomal positions (X-axis) and the observed -$\log_{10}$(P-values) in the meta-analyses (Y-axis). All SNPs on each chromosome are shown in the same colour but a distinct colour from that of the adjacent chromosome. The horizontal line in the Figures indicate the genome-wide significance level (-$\log_{10}$P-value=7.30). Q-Q plots on the right: The X-axis shows the expected distribution of -$\log_{10}$(P-values) under the null hypothesis of no association. The Y-axis shows the distribution of the observed -$\log_{10}$(P-values) in the meta-analyses. The red indicator lines show where X=Y. Genomic inflation factor lambda (λ) has been indicated for each analysis separately.

A) OAG. λ=1.006.

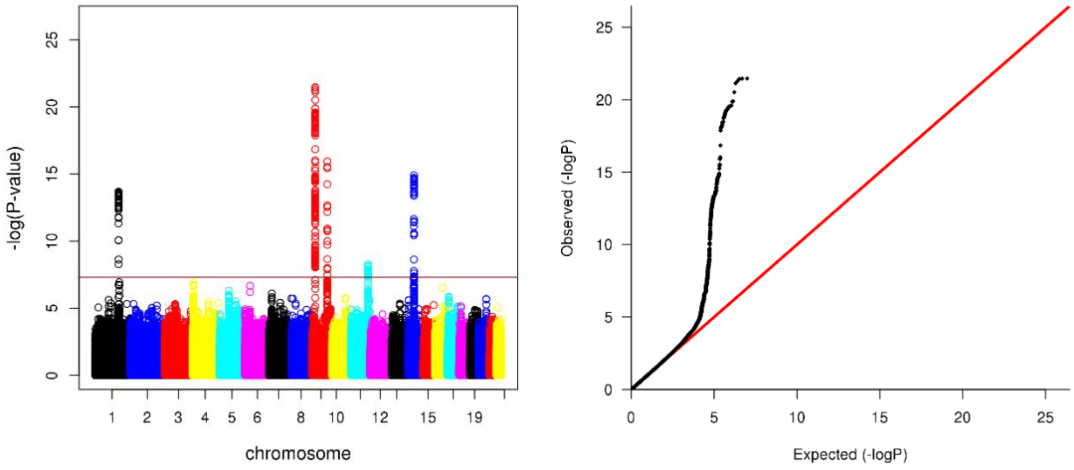

B) OAG + VCDR (European ancestry). λ= 1.046.

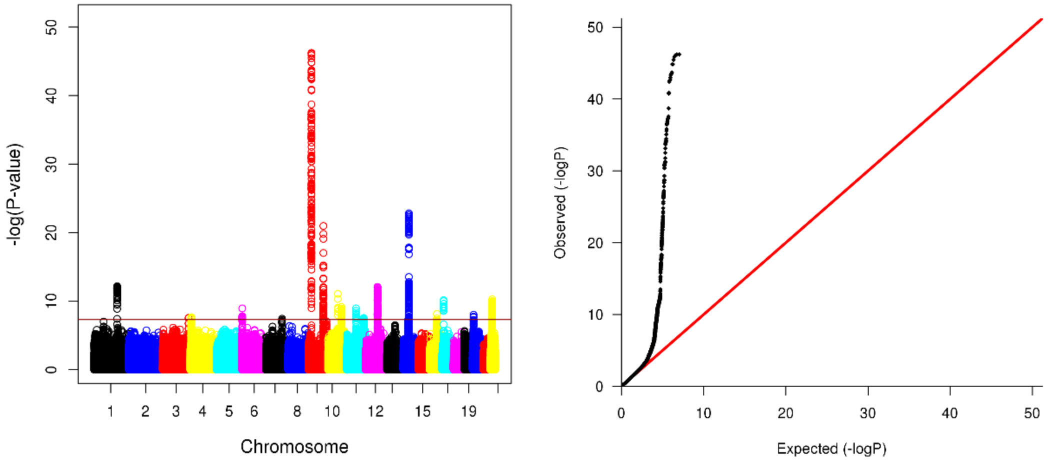

C) OAG + VCDR (Asians + European ancestry). λ= 1.052.

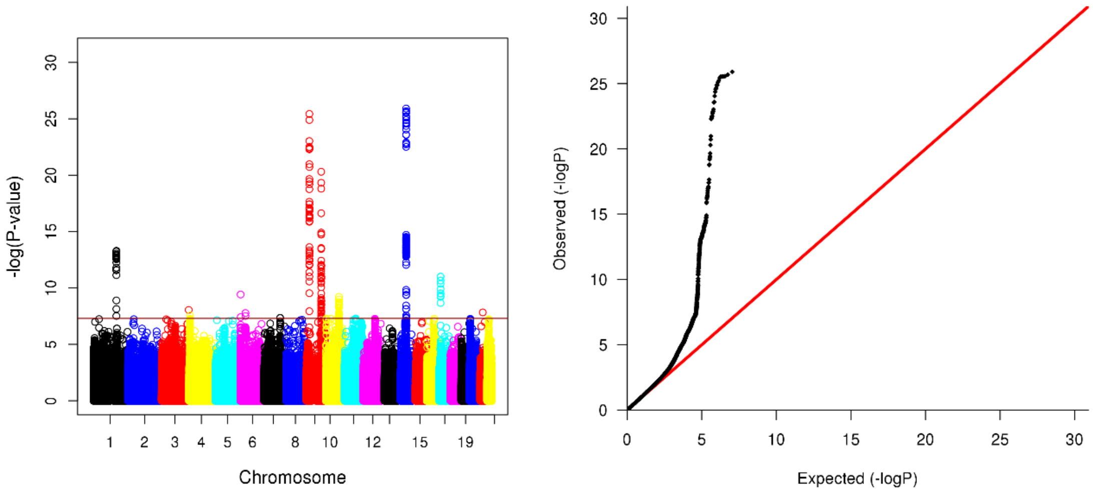

D) OAG + VCDR (Asians). λ= 1.045.

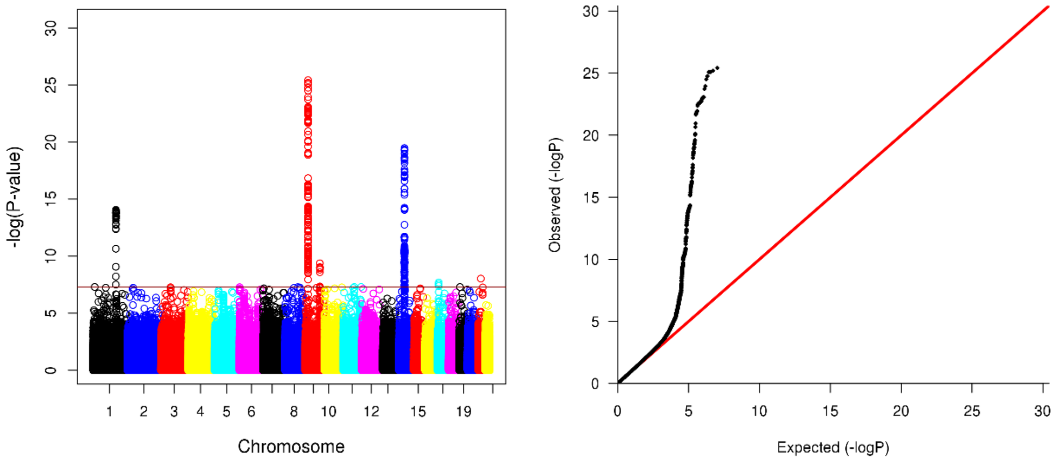

E) OAG + CA (European ancestry). λ=1.043

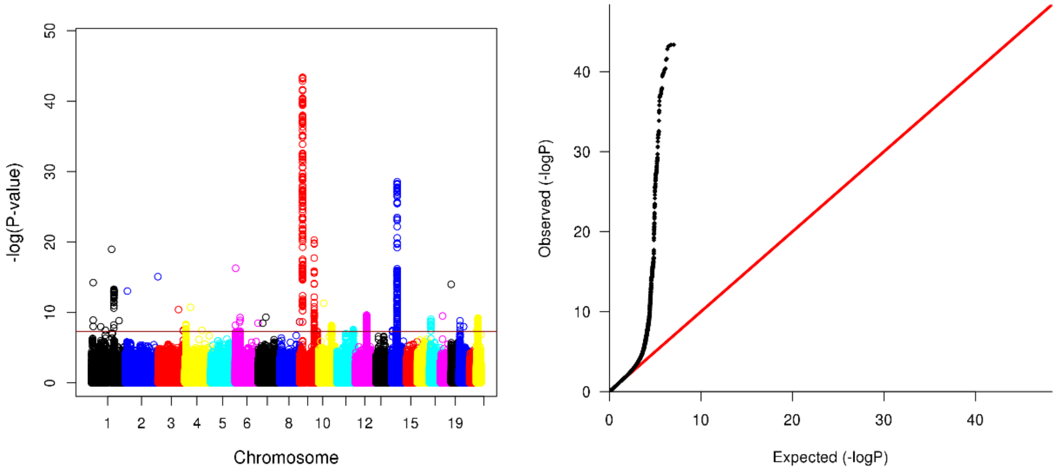

F) OAG + CA (European ancestry + Asians). λ= 1.045.

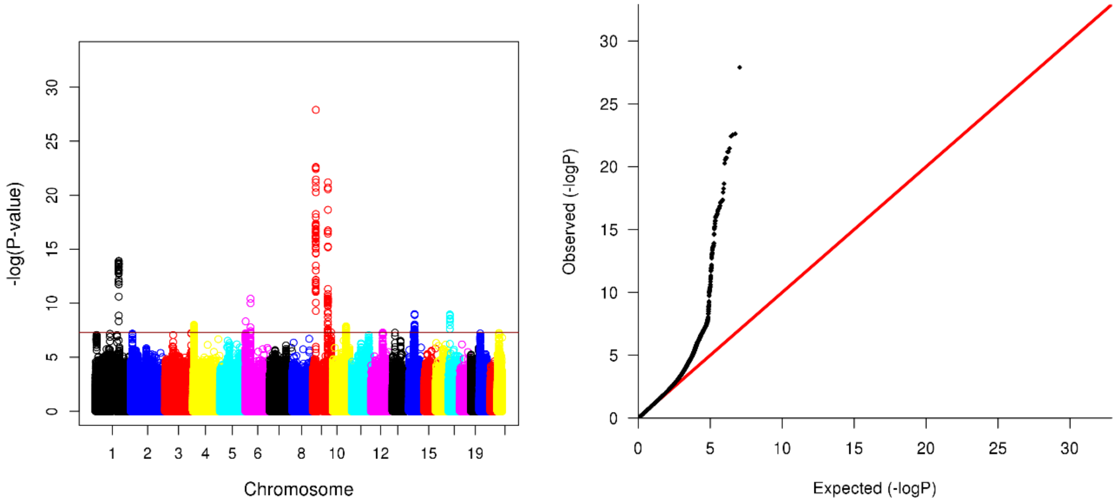

G) OAG + CA (Asians). λ= 1.035.

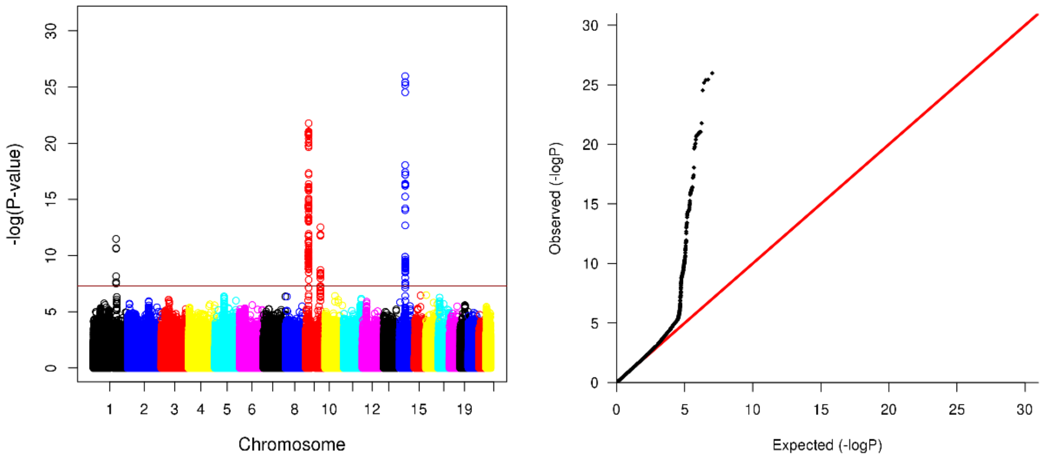

H) OAG + DA (European ancestry). λ=1.039.

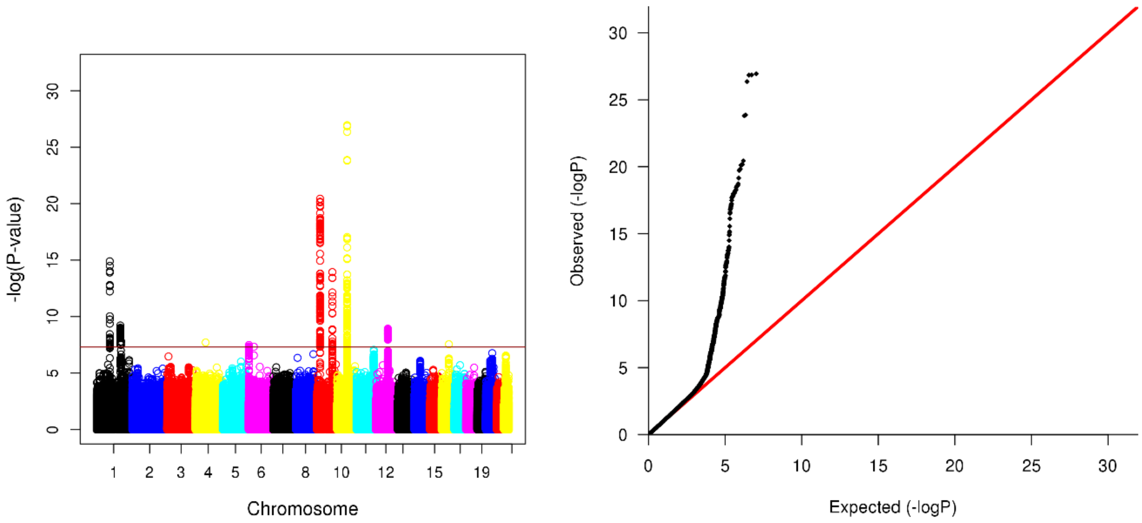

I) OAG + DA (European ancestry + Asians). λ=1.041.

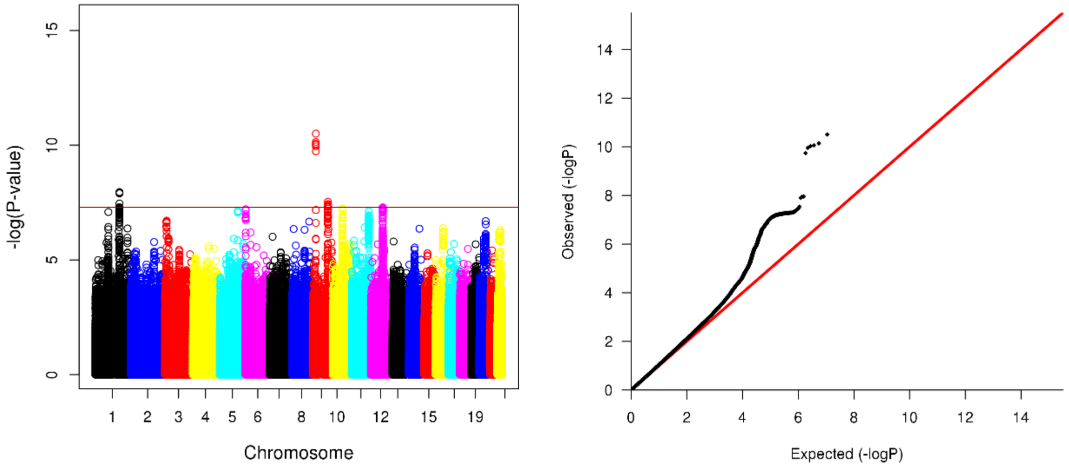

J) OAG + DA (Asians). λ= 1.035.

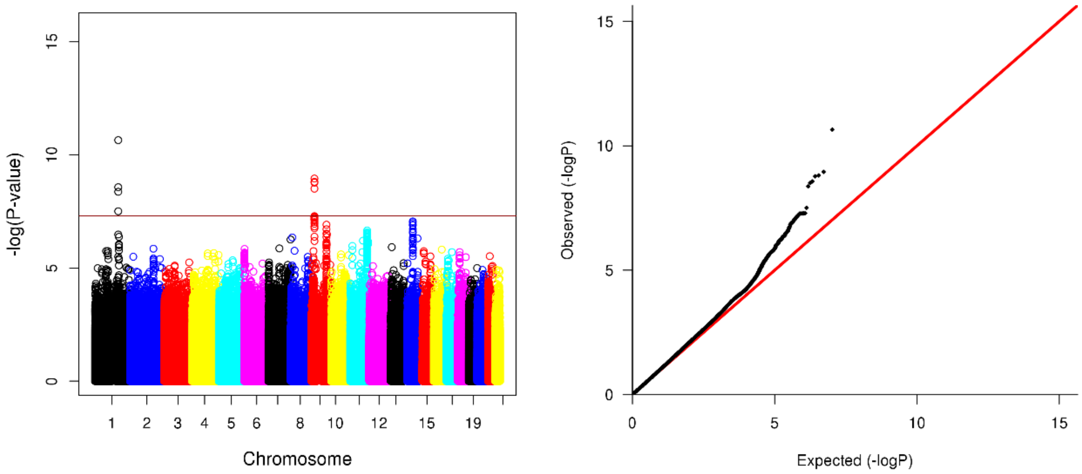

K) OAG + IOP (European ancestry). λ= 1.039.

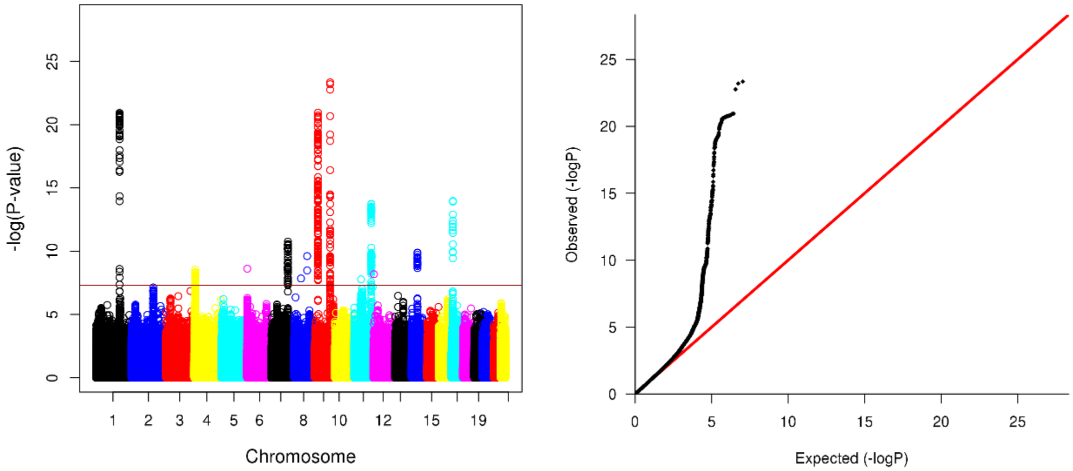

L) OAG + IOP (European ancestry +Asians). λ= 1.040.

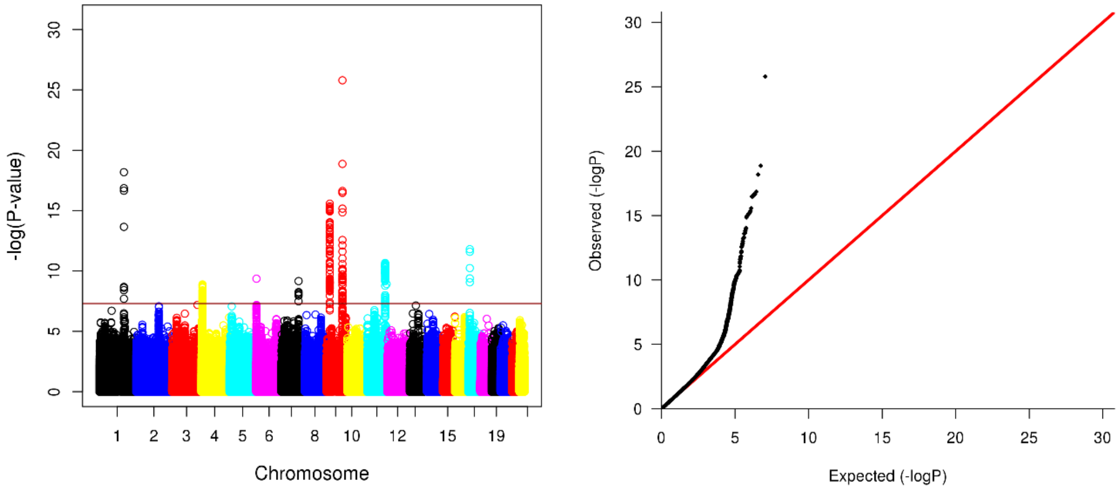

M) OAG + IOP (Asians). λ= 1.033.

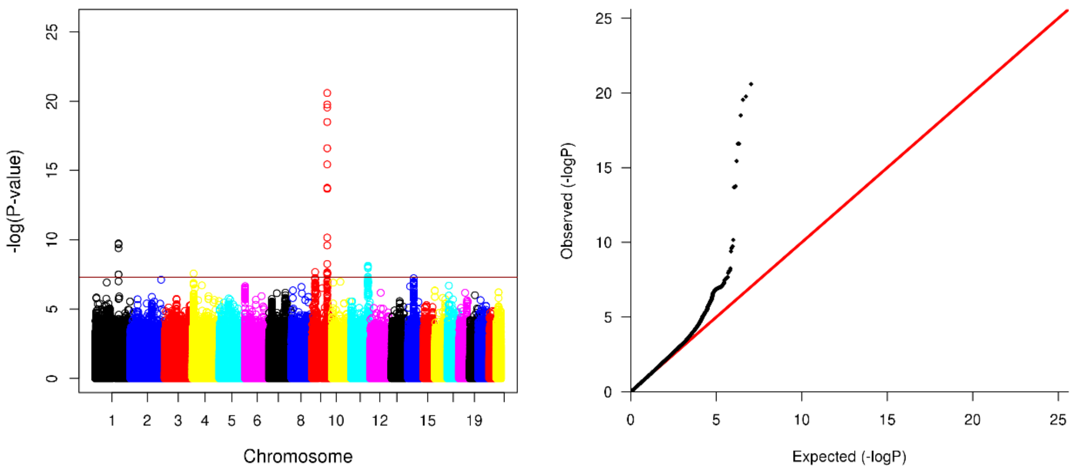

Supplementary Figure 2. This figure shows the expression levels of the nearest genes (Y-axis) to the best associated SNPs within the new OAG risk loci in relevant eye tissues (X-axis). The expression levels are reported in mean count per million reads (as per color key) from RNA sequencing data (see the methods section for more details).

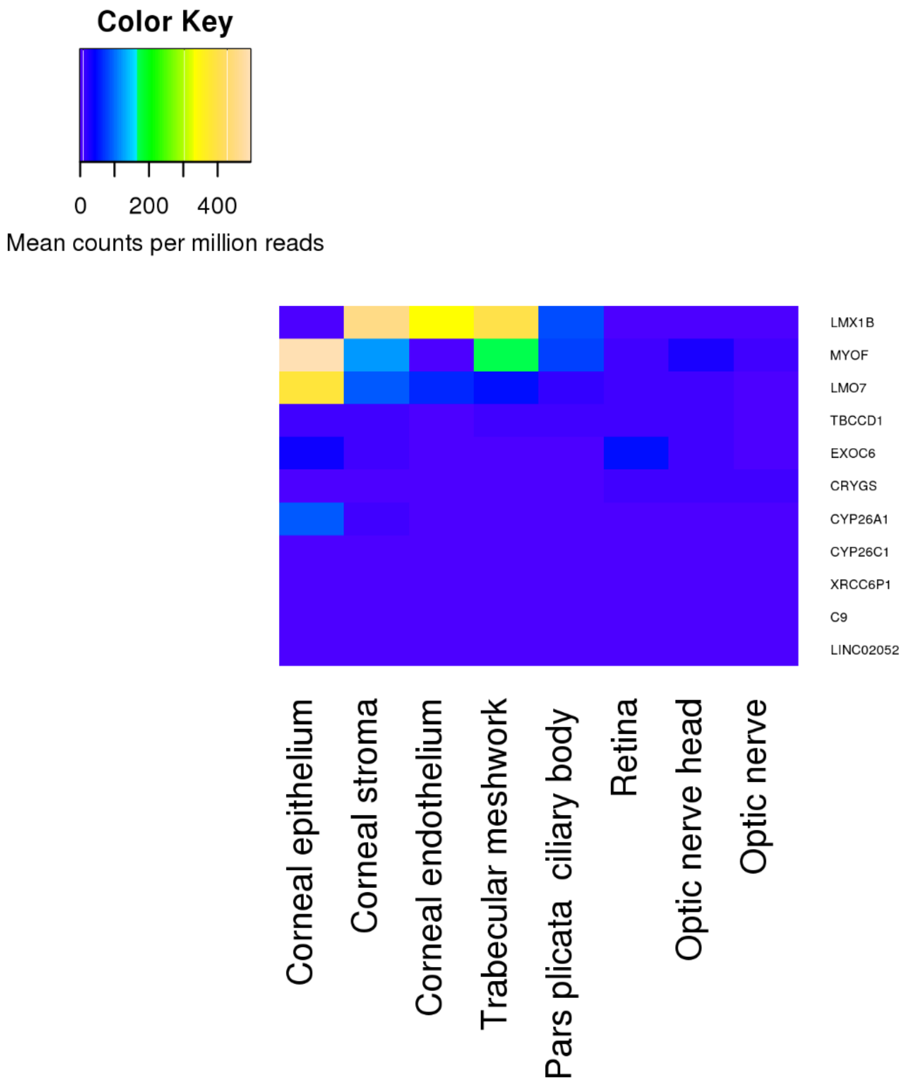

Supplementary Discussion:

In this section we have discussed the bioinformatics functional annotations for the top loci identified in this study.

rs72815193: Several SNPs in high LD (LD r^2 >0.80) with rs72815193 are located in regions marked by active histone modifications (promoter or enhancer histone marks) and DNaseI hypersensitivity in various tissues[10]. In addition, rs56871408 and rs6583867, the two SNPs in high LD with rs72815193, are bound by transcriptional regulators FOXA1 and GATA3, respectively. rs72815193 and a majority of the high LD SNPs modify the predicted sequence motifs for binding of many protein binding sites; for example, rs72815193 changes the motif for binding of *NRSF*. In addition, rs72815193 is an eQTL for *XRCC6P1* in subcutaneous adipose tissue ($P = 3.8 \times 10^{-6}$) in the GTEx dataset[11] with posterior probability of 1 that an eQTL exist in each tissue tested in the cross-tissue meta-analysis[12,13].

rs56962872: This SNP and its highly correlated variant rs6151152 (LD r^2=1 with rs56962872) change the sequence motif for binding of several proteins. rs6151152 is located within a region with an enhancer histone mark (H3K4me1; the monomethylation of the 4th residue (a lysine) from the N-terminal of the H3 protein) in several cell lines or tissues.

rs6478746: resides within a region with an enhancer histone mark in several cells and tissues, and region of DNaseI hypersensitivity in fetal muscle. rs4837100, the SNP in high LD with this variant, is an eQTL ($P = 7 \times 10^{-5}$) for *LMX1B* in sub-cutaneous adipose tissues in the GTEx dataset, and an eQTL ($P = 5.89 \times 10^{-6}$) for *FAM125B* in whole blood in the Blood eQTL Browser. rs6478746 changes the sequence motif for binding of transcription activator, Nrf-2.

rs9530458: This SNP and several SNPs in high LD (LD r^2 >0.80) reside within promoter and enhancer histone markers and DNaseI hypersensitivity sites for many tissues. In addition, these SNPs modify the sequence motif for binding of many proteins.

Transcriptional regulators such as CTCF and IRF4 bind to the variants in high LD with rs9530458, and thus these SNPs may play a role in regulation of transcription of the relevant genes.

Acknowledgment:

The NEIGHBORHOOD consortium:
R Rand Allingham[18], Murray Brilliant[19], Donald L. Budenz[20], John H. Fingert[21,22], Douglas Gaasterland[23], Teresa Gaasterland[24], Lisa Hark[25], Michael Hauser[18,26], Robert P Igo Jr[27], Peter Kraft[28,29], Richard K. Lee[30], Paul R. Lichter[31], Yutao Liu[32,33], Syoko Moroi[31], Margaret Pericak-Vance[34], Anthony Realini[35], Doug Rhee[36], Julia E. Richards[31,37], Robert Ritch[38], Joel S. Schuman[39], William K. Scott[34], Kuldev Singh[40], Arthur J. Sit[41], Douglas Vollrath[42], Gadi Wollstein[39], Donald J. Zack[43].

[18]Department of Ophthalmology Duke University Medical Center, Durham, NC; [19]Center for Human Genetics, Marshfield Clinic Research Foundation, Marshfield, WI; [20]Department of Ophthalmology, University of North Carolina, Chapel Hill, NC; [21]Department of Ophthalmology University of Iowa, College of Medicine, Iowa City, IO; [22]Department of Anatomy and Cell Biology, University of Iowa, College of Medicine, Iowa City, IO; [23]Eye Doctors of Washington, Chevy Chase, MD; [24]Scripps Genome Center, University of California at San Diego, San Diego, CA; [25]Wills Eye Hospital, Glaucoma Research Center, Philadelphia, PA, USA; [26]Department of Medicine, Duke University Medical Center, Durham, NC; [27]Department of Epidemiology and Biostatistics, Institute for Computational Biology, Case Western Reserve University School of Medicine, Cleveland, Ohio, USA; [28]Department of Epidemiology, Harvard School of Public Health, Boston, MA; [29]Program in Genetic Epidemiology and Statistical Genetics, Harvard School of Public Health, Boston, MA; [30]Bascom Palmer Eye Institute University of Miami Miller School of Medicine, Miami, FL; [31]Department of Ophthalmology and Visual Sciences, University of Michigan, Ann Arbor, MI; [32]Department of Cellular Biology and Anatomy, Georgia Regents University, Augusta, GA; [33]James & Jean Culver Vision Discovery Institute, Georgia Regents University,

Augusta, GA; [34]Institute for Human Genomics, University of Miami Miller School of Medicine, Miami, FL; [35]Department of Ophthalmology, West Virginia University Eye Institute, Morgantown, WV; [36]Department of Ophthalmology and Visual Sciences, UH Cleveland Medical Center; Cleveland, OH; [37]Department of Epidemiology, University of Michigan, Ann Arbor, MI; [38]Einhorn Clinical Research Center, Department of Ophthalmology, New York Eye and Ear Infirmary of Mt. Sinai, New York, NY; [39]Department of Ophthalmology, University of Pittsburgh, Pittsburgh, PA; [40]Department of Ophthalmology, Stanford University School of Medicine, Palo Alto, CA; [41]Department of Ophthalmology, Mayo Clinic, Rochester, MN; [42]Department of Genetics, Stanford University School of Medicine, Palo Alto, CA; [43]Wilmer Eye Institute, Johns Hopkins University Hospital, Baltimore, MD

References:

1 Wiggs, J. L. *et al.* Common variants at 9p21 and 8q22 are associated with increased susceptibility to optic nerve degeneration in glaucoma. *PLoS Genet* **8**, e1002654, doi:10.1371/journal.pgen.1002654 (2012).

2 Wiggs, J. L. *et al.* The NEIGHBOR consortium primary open-angle glaucoma genome-wide association study: rationale, study design, and clinical variables. *J Glaucoma* **22**, 517-525, doi:10.1097/IJG.0b013e31824d4fd8 (2013).

3 Bailey, J. N. *et al.* Genome-wide association analysis identifies TXNRD2, ATXN2 and FOXC1 as susceptibility loci for primary open-angle glaucoma. *Nat Genet* **48**, 189-194, doi:10.1038/ng.3482 (2016).

4 Feuer, W. J. *et al.* The Ocular Hypertension Treatment Study: reproducibility of cup/disk ratio measurements over time at an optic disc reading center. *Am J Ophthalmol* **133**, 19-28 (2002).

5 Price, A. L. *et al.* Principal components analysis corrects for stratification in genome-wide association studies. *Nat Genet* **38**, 904-909, doi:10.1038/ng1847 (2006).

6 Aulchenko, Y. S., Struchalin, M. V. & van Duijn, C. M. ProbABEL package for genome-wide association analysis of imputed data. *BMC Bioinformatics* **11**, 134, doi:10.1186/1471-2105-11-134 (2010).

7 Willer, C. J., Li, Y. & Abecasis, G. R. METAL: fast and efficient meta-analysis of genomewide association scans. *Bioinformatics* **26**, 2190-2191, doi:10.1093/bioinformatics/btq340 (2010).

8 Gharahkhani, P. *et al.* Common variants near ABCA1, AFAP1 and GMDS confer risk of primary open-angle glaucoma. *Nat Genet* **46**, 1120-1125, doi:10.1038/ng.3079 (2014).

9 Springelkamp, H. *et al.* New insights into the genetics of primary open-angle glaucoma based on meta-analyses of intraocular pressure and optic disc characteristics. *Hum Mol Genet* **26**, 438-453, doi:10.1093/hmg/ddw399 (2017).

10 Ward, L. D. & Kellis, M. HaploReg: a resource for exploring chromatin states, conservation, and regulatory motif alterations within sets of genetically linked variants. *Nucleic Acids Res* **40**, D930-934, doi:10.1093/nar/gkr917 (2012).

11 Lonsdale, J. *et al.* The Genotype-Tissue Expression (GTEx) project. *Nat Genet* **45**, 580-585, doi:10.1038/ng.2653 (2013).
12 Han, B. & Eskin, E. Random-effects model aimed at discovering associations in meta-analysis of genome-wide association studies. *Am J Hum Genet* **88**, 586-598, doi:10.1016/j.ajhg.2011.04.014 (2011).
13 Han, B. & Eskin, E. Interpreting meta-analyses of genome-wide association studies. *PLoS Genet* **8**, e1002555, doi:10.1371/journal.pgen.1002555 (2012).
